# Characteristics of the 6th Japanese wave of COVID-19 in hemodialysis patients

**DOI:** 10.1186/s41100-022-00451-2

**Published:** 2022-12-02

**Authors:** Munenori Haruta, Shigeru Otsubo, Yuriko Otsubo

**Affiliations:** 1 Department of Clinical Engineering, Faculty of Human Care at Makuhari, Tohto University, 1-1 Hibino, Mihama-Ward, Chiba-City, Chiba-Prefecture 261-0021 Japan; 2 Department of Nephrology, Sangenjaya Hospital, Tokyo, Japan

**Keywords:** COVID-19, Hemodialysis, Omicron variant, SARS-CoV-2, 6th Japanese wave

## Abstract

**Background:**

We examined the clinical characteristics of hemodialysis patients with COVID-19 during the 6th wave of infection (mainly Omicron variant) in Japan.

**Methods:**

Hemodialysis patients admitted in January 2022 and thereafter were grouped as the 6th wave group (*n* = 53), while others were grouped as the 1st–5th wave group (*n* = 47).

**Results:**

The proportion of vaccinations was significantly higher in the 6th wave group than in the 1st–5th wave group (96.2% vs 10.6%, *p* < 0.0001). Neutralizing antibody and molnupiravir were used more frequently in the 6th wave group (75.5% and 88.7%) than in the 1st–5th wave group (14.9% and 0%, both *p* < 0.0001). The critical disease was seen in 21.3% of the patients in the 1st–5th wave group and 0% in the 6th wave group (*p* < 0.001).

**Conclusion:**

The prognosis of hemodialysis patients in the 6th wave group was good. The vaccination and advances in the treatment may have contributed to the outcomes.

## Background

An outbreak of coronavirus disease 2019 (COVID-19), now known to be caused by severe acute respiratory syndrome coronavirus 2 (SARS-CoV-2), that began in China in December 2019 has since spread rapidly throughout the world [1]. Different variants of SARS-CoV-2 have been identified, such as the Delta variant. The Omicron variant is a new, heavily mutated SARS-CoV-2 variant that was designated as a variant of concern by the World Health Organization on November 26, 2021 [2]. The first confirmed Omicron variant was reported to WHO on November 24, 2021, from a sample collected in South Africa [3]. In the general population in England, Omicron cases were reported to have a reduced risk of hospitalization, compared with Delta [4]. To date, Japan has experienced 6 waves of infection, with the 6th wave beginning in January 2021 and mainly consisting of cases infected with the Omicron variant. In addition, vaccination against SARS-CoV2 has now become widespread in Japan, and new medicines, such as neutralizing antibodies or antiviral agents, have been developed and are being used clinically.

On the other hand, hemodialysis patients are at a high risk for the development of severe COVID-19, since factors identified as risk factors for severe COVID-19 are often present in these patients including old age, hypertension, cardiovascular comorbidity, and underlying diabetes mellitus. In addition, these patients also show impaired antiviral immune responses because of their impaired kidney functions. Hemodialysis patients with COVID-19 reportedly have a poor prognosis [5–9]. In the present study, we examined the clinical characteristics of hemodialysis patients with COVID-19 during the 6th wave of infection at our hospital.

## Methods

A total of 106 hemodialysis patients were admitted to our hospital for the treatment of COVID-19 between April 2020 and May 2022; after the exclusion of 6 patients who were transferred to our hospital after receiving treatment for the acute disease phase, the remaining 100 hemodialysis patients were retrospectively enrolled in this study. We divided the patients according to their hospitalization dates. Patients who were admitted in January 2022 and thereafter were grouped as the 6th wave group (*n* = 53), while those admitted before January 2022 were grouped as the 1st–5th wave group (*n* = 47). The diagnosis of COVID-19 was made by the reverse transcription polymerase chain reaction (RT-PCR) test or the antigen test for SARS-CoV-2. Treatments were performed in accordance with the Clinical Practice Guide for the Diagnosis and Treatment of COVID-19 at that time [10]. Patients received symptomatic treatment for various symptoms, such as fever and cough. For 5th group patients, Casirivimab/Imdevimab were used, if possible. Similarly, for 6th group patients, Sotrovimab and Molnupiravir were used, if possible. Remdesivir was first not recommended for patients with severe renal impairment. However, it now became commonly used also in dialysis patients, so we considered using it for 5th and 6th wave group patients when they tend to develop severe outcomes. Dexamethasone was usually used for severe cases. In patients with elevated serum D-dimer levels, the indication for anticoagulant therapy was determined by taking into consideration the risk of bleeding. The patients’ clinical data, including the symptoms and results of laboratory and other examinations and the clinical outcomes, were collected retrospectively from the medical records. The disease severity in the patients was categorized as mild, moderate, severe, or critical. The mild disease was defined as a lack of respiratory symptoms and an SpO2 ≥ 96%. The moderate disease was defined as mild respiratory symptoms, radiological evidence of pneumonia, and 93% < SpO2 < 96%. Severe disease was defined as SpO2 ≤ 93% and the use of oxygen support or the initiation of steroid therapy. The critical disease was defined as an SpO2 remaining at or below 93% despite oxygen supplementation at 5 L/min or more via a face mask or death. We compared various clinical findings between the 1st–5th wave group and the 6th wave group. In the general course of COVID-19, pneumonia can begin to worsen and reach critical severity about 7–10 days after clinical onset [10]. So, the relationship between the maximum C-reactive protein (CRP) value within 7 days after clinical onset and disease severity was investigated for cases in which the necessary data were available.

Data were expressed as the means ± standard deviation or medians (interquartile ranges). The statistical significance of the differences was determined using a two-sided, paired *t* test. For non-normally distributed variables, a two-sided Wilcoxon signed-rank test was used. The chi-square or Fisher exact probability test was used for categorical data. A receiver operating characteristic (ROC) curve analysis was used to determine the optimum cutoff point for the maximum CRP value within 7 days after clinical onset for predicting a critical (including death) outcome. All the statistical calculations were performed using JMP 5.1 software. A *p* value of less than 0.05 was considered as being statistically significant. This study was conducted in accordance with the principles of the Declaration of Helsinki and with the approval of the research ethics committee of our Hospital (Approved No. R0206).

## Results

Table 1 compares the characteristics of hemodialysis patients in the 1st–5th wave group and those in the 6th wave group. Sex, age, dialysis vintage, the number of days from onset until diagnostic testing, and the number of days from onset until hospitalization were not statistically different between the two groups. The most common cause of end-stage kidney disease was diabetic nephropathy in both groups. The complication rate, such as diabetes mellitus or ischemic heart disease, dry weight, and body mass index also showed no significant differences between the two groups. The proportion of vaccinations was significantly higher in the 6th wave group than in the 1st–5th wave group (0 dose: 3.8% vs. 89.4%, *p* < 0.0001; more than one dose: 96.2% vs. 10.6%, *p* < 0.0001; 2 doses or more: 96.2% vs. 8.5%, *p* < 0.0001; 3 doses: 18.9% vs. 0%, *p* = 0.001).

**Table 1:** Comparison of characteristics and laboratory data between the 1st–5th wave group and the 6th wave group

	1st–5th wave group	6th wave group	*p* values
Gender (M/F)	38/9	44/9	NS
Age (year)	59.7 ± 12.9	63.6 ± 12.8	NS
Dialysis vintage (year)	6.6 ± 6.0	8.2 ± 7.8	NS
Days from onset to test for diagnosis (days)	1.3 ± 2.3	0.9 ± 1.2	NS
Days from onset to hospitalization (days)	3.4 ± 2.8	3.0 ± 2.4	NS
Primary cause of ESKD, *n* (%)			
Chronic glomerulonephritis	10 (21.3)	13 (24.5)	NS
Diabetic nephropathy	21 (44.7)	21 (39.6)	NS
Nephrosclerosis	5 (10.6)	8 (15.1)	NS
Polycystic kidney disease	2 (4.3)	7 (13.2)	NS
Unknown and others	9 (19.1)	4 (7.5)	NS
Complication, *n* (%)			
Diabetes mellitus	22 (46.8)	22 (41.5)	NS
Ischemic heart disease	5 (7.5)	11 (8.5)	NS
Cerebrovascular disease	8 (17.0)	5 (9.4)	NS
Chronic respiratory disease	1 (2.1)	0 (0)	NS
Critical limb ischemia	5 (10.6)	5 (9.4)	NS
Hypertension	46 (97.9)	50 (94.3)	NS
Dry weight (kg)	64.8 ± 17.7	64.4 ± 15.4	NS
Body mass index (kg/m^2^)	23.2 ± 4.9	23.6 ± 4.7	NS
Number of vaccinations, *n* (%)			
0 dose	42 (89.4)	2 (3.8)	< 0.0001
More than one dose	5 (10.6)	51 (96.2)	< 0.0001
2 doses (primary series) or more	4 (8.5)	51 (96.2)	< 0.0001
3 doses (booster)	0 (0)	10 (18.9)	0.001
Symptom at admission, *n* (%)			
Cough	29 (61.7)	37 (69.8)	NS
Sore throat	5 (10.6)	32 (60.4)	< 0.0001
Headache	12 (25.5)	5 (9.4)	0.032
Nasal discharge	8 (17.0)	14 (26.4)	NS
Nausea/vomiting	8 (17.0)	3 (5.7)	NS
Diarrhea	8 (17.0)	4 (7.5)	NS
Olfactory and taste abnormalities	5 (10.6)	3 (5.7)	NS
Blood sample			
Days from the onset to blood sampling (days)	4.4 ± 2.5	3.2 ± 2.6	0.024
Total protein (g/dL)	6.4 ± 0.6	6.7 ± 0.8	0.029
Albumin (g/dL)	3.3 ± 0.4	3.6 ± 0.5	0.007
Aspartate aminotransferase (U/L)	21 ± 10	19 ± 12	NS
Alanine aminotransferase (U/L)	14 ± 8	15 ± 11	NS
Lactate dehydrogenase (U/L)	285 ± 83	238 ± 70	0.003
Creatine kinase (U/L)	184 ± 191	139 ± 136	NS
Urea nitrogen (mg/dL)	64.1 ± 20.5	55.8 ± 17.2	0.032
Creatinine (mg/dL)	11.9 ± 4.4	10.5 ± 3.8	NS
Ferritin (ng/ml)	286.6 ± 280.92	265.4 ± 257.3	NS
C-reactive protein (mg/dL)*	6.10 (1.18–10.43)	1.98 (0.55–5.21)	0.001
Hemoglobin (g/dL)	11.2 ± 1.5	11.1 ± 1.6	NS
White blood cells (/μL)	5000 ± 2000	5200 ± 2500	NS
Lymphocytes (%)	15.9 ± 7.8	17.9 ± 7.3	NS
Lymphocytes (/μL)	750 ± 370	860 ± 350	NS
Platelet (×104/μL)	15.1 ± 4.4	16.7 ± 7.6	NS
D-dimer (μg/mL)	2.8 ± 3.9	4.2 ± 10.6	NS
Maximum CRP level during hospitalization (mg/dL)*	10.42 (5.22–14.12)	1.99 (0.57–6.24)	< 0.0001

*ESKD* End-stage kidney disease; *CRP* C-reactive protein

*Median (interquartile range) level of CRP

The sore throat was a more frequent symptom in the 6th wave group (60.4%) than in the 1st–5th wave group (10.6%, *p* < 0.0001). Headache was a less frequent symptom in the 6th wave group (9.4%) than in the 1st–5th wave group (25.5%, *p* = 0.032). The number of days from onset until blood sampling was significantly shorter in the 6th wave group (3.2 ± 2.6 days) than in the 1st–5th wave group (4.4 ± 2.5 days, *p* = 0.024). The serum levels of total protein (TP) (*p* = 0.024), albumin (*p* = 0.007), lactate dehydrogenase (LDH) (*p* = 0.003), urea nitrogen (*p* = 0.032), and CRP (*p* = 0.001) were lower in the 6th wave group than in the 1st–5th wave group. The maximum CRP level during hospitalization was lower in the 6th wave group [1.99 (0.57–6.24) (5.28 ± 8.50) mg/dL] than in the 1st–5th wave group [10.42 (5.22–14.12) (10.9 ± 8.31) mg/dL, *p* < 0.0001].

Table 2 shows the treatment for COVID-19. Neutralizing antibody therapy was used more frequently in the 6th wave group (75.5%) than in the 1st–5th wave group (14.9%, *p* < 0.0001). Casirivimab and imdevimab were used only in the 1st–5th wave group, and sotrovimab was used only in the 6th wave group as an antibody therapy. Molnupiravir was also used more frequently in the 6th wave group (88.7%) than in the 1st–5th wave group (0%, *p* < 0.0001). There was no difference in the frequency of use of remdesivir between the two groups. Dexamethasone and heparinization were more frequently used in the 1st–5th wave group (53.2% and 46.8%, respectively) than in the 6th wave group (5.7%, *p* < 0.0001 and 13.2%, *p* < 0.001, respectively).

**Table 2:** Comparison of the treatment for COVID-19 between the 1st–5th wave group and the 6th wave group

Treatment, *n* (%)	1st–5th wave group	6th wave group	*p* values
Neutralizing antibody therapy	7 (14.9)	40 (75.5)	< 0.0001
Casirivimab/Imdevimab	7 (14.9)	0 (0)	0.004
Sotrovimab	0 (0)	40 (75.5)	< 0.0001
Antiviral therapy			
Molnupiravir	0 (0)	47 (88.7)	< 0.0001
Remdesivir	6 (12.8)	12 (22.6)	NS
Immunosuppressive therapy			
Dexamethasone	25 (53.2)	3 (5.7)	< 0.0001
Anticoagulation therapy			
Heparinization	22 (46.8)	7 (13.2)	< 0.001

Table 3 shows the clinical outcomes. The maximum body temperature was significantly higher in the 1st–5th wave group (38.8 ± 0.8 °C) than in the 6th wave group (38.1 ± 0.9 °C, *p* < 0.0001). Only one patient (9.1%) had no fever during hospitalization in the 1st–5th wave group, while 12 patients (22.6%) had no fever during hospitalization in the 6th wave group (*p* = 0.002). Mild to moderate disease was seen in 34.0% of the patients in the 1st–5th wave group and 69.8% in the 6th wave group (*p* < 0.001). Severe disease was seen in 44.7% of the patients in the 1st–5th wave group and 30.2% in the 6th wave group. Critical disease (including death) was seen in 21.3% of the patients in the 1st–5th wave group and 0% in the 6th wave group (*p* < 0.001). Death occurred in 8.5% of the patients in the 1st–5th wave group and 0% in the 6th wave group (*p* = 0.046). The number of days from onset until hospital discharge was 18.1 ± 7.2 days in the 1st–5th wave group, which was longer than that in the 6th wave group (11.5 ± 3.3 days, *p* < 0.0001). When the number of days until a reduction in temperature occurred was compared (excluding patients who used dexamethasone and those who did not have a fever during the clinical course), the time period was significantly longer in the 1st–5th wave group (9.5 ± 3.0 days, *n* = 15) than in the 6th wave group (6.1 ± 4.6 days, *n* = 41, *p* = 0.012).

**Table 3:** Comparison of the clinical outcomes between the 1st–5th wave group and the 6th wave group

	1st–5th wave group	6th wave group	*p* values
Maximum body temperature	38.8 ± 0.8	38.1 ± 0.9	< 0.0001
No fever during hospitalization	1 (9.1)	12 (22.6)	0.002
Clinical outcomes			
Mild to moderate	16 (34.0)	37 (69.8)	< 0.001
Severe	21 (44.7)	16 (30.2)	NS
Critical (including death)	10 (21.3)	0 (0)	< 0.001
Death	4 (8.5)	0 (0)	0.046
The days from onset to discharge	18.1 ± 7.2	11.5 ± 3.3	< 0.0001

**Fig. 1:**
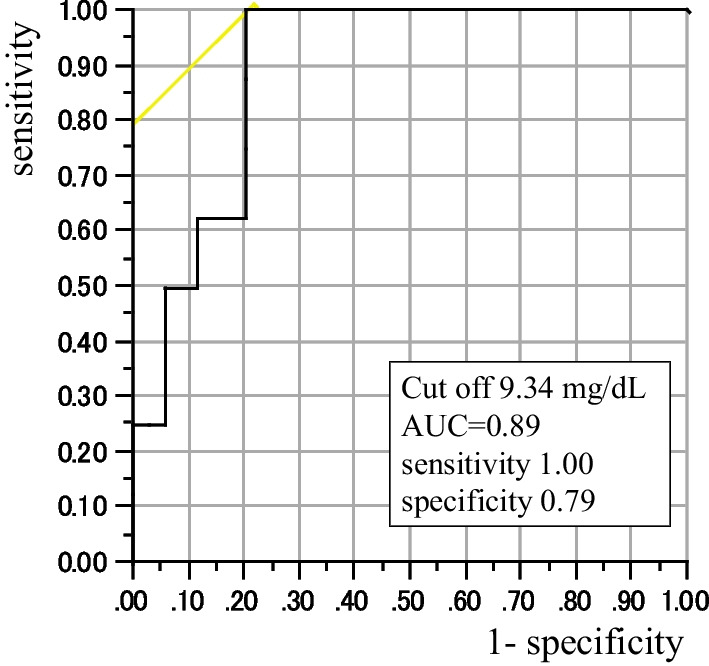
Results for the ROC of the maximum CRP values within 7 days after clinical onset in the 1st–5th wave group as a predictor of critical (including death) outcomes. The best cutoff point was 9.34 mg/dL, with a sensitivity of 100% and a specificity of 79%. The area under the ROC curve was 0.89. *AUC* Area under the curve

The maximum CRP values within 7 days after clinical onset were compared between the two groups. The CRP levels were significantly higher in the 1st–5th wave group [8.14 (2.84–11.24) mg/dL, *n* = 42] than in the 6th wave group [1.99 (0.59–6.00) mg/dL, *n* = 51, *p* < 0.001]. Figure 1 shows the results for the ROC of the maximum CRP values within 7 days after clinical onset in the 1st–5th wave group as a predictor of critical (including death) outcomes. The best cutoff point was 9.34 mg/dL, with a sensitivity of 100% and a specificity of 79%. The area under the ROC curve was 0.89.

**Fig. 2:**
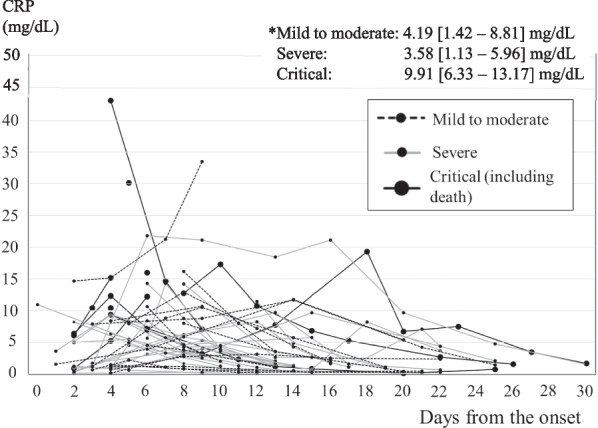
Time course of the changes in the serum CRP level in the 1st–5th wave group. In all the critical cases in the 1st–5th wave group, the serum CRP level exceeded 9.34 mg/dL within 7 days after clinical onset

**Fig. 3:**
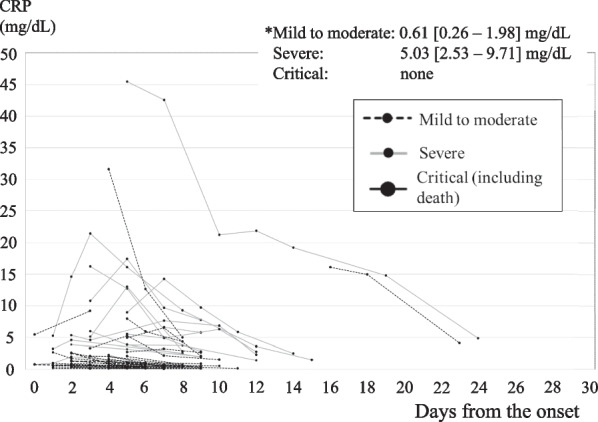
Time course of the changes in the serum CRP level in the 6th wave group. Seven hemodialysis patients in the 6th wave group had CRP values above 9.34 mg/dL within 7 days after clinical onset, which was the cutoff for predicting critical (including death) outcomes, but none of these patients developed critical outcomes (1 moderate, 6 severe)

Figures 2 and 3 show the time course of the changes in the serum CRP level. The large black circles and black lines represent cases with critical disease, the gray-black lines represent cases with severe disease, and the dotted lines represent cases with mild to moderate disease. In the 1st–5th wave group, 6 patients had a serum CRP level of more than 9.34 mg/dL within 7 days after clinical onset but did not develop critical outcomes (Fig. 2). Three of them had been vaccinated at least once, and 3 of them had received anti-neutralizing antibody therapy. Only two of the 6 patients had not received either a vaccine or neutralizing antibody therapy. In all the critical cases in the 1st–5th wave group, the serum CRP level exceeded 9.34 mg/dL within 7 days after clinical onset. On the other hand, 7 hemodialysis patients in the 6th wave group had CRP values above 9.34 mg/dL within 7 days after clinical onset, but none of these patients developed critical outcomes (1 moderate, 6 severe, Fig. 3).

## Discussion

A larger proportion of hemodialysis patients affected by COVID-19 during the 6th wave had been vaccinated (2 doses or more, 96.2%; 3 doses, 18.9%), compared with patients during the 1st–5th waves. Sore throats were more common (60.4%), and headaches (9.4%) and fever during hospitalization (77.4%) were less common in the 6th wave group. More of the patients in the 6th wave group received neutralizing antibody (75.5%) and molnupiravir (88.7%), compared with the 1st–5th wave group. Fewer patients required steroid (5.7%) or anticoagulant (13.2%) therapy in the 6th wave group. The time period from onset until a reduction in fever (6.1 ± 4.6 days) and the time period from onset until hospital discharge (11.5 ± 3.3 days) were relatively short in the 6th wave group. Overall, the patient outcomes in the 6th wave group were good (mild to moderate, 69.8%; severe, 30.2%; critical (including death), 0%; death, 0%). Their maximum CRP level was relatively low [1.99 (0.57–6.24) mg/dL], and even if their CRP level increased above 9.34 mg/dL, which was the cutoff for predicting critical (including death) outcomes, they did not develop critical disease.

The Omicron variant accounted for the majority of the 6th wave of COVID-19 in Japan. In the general population, a sore throat was more likely to be reported by subjects infected with Omicron (53% of Omicron cases, 34% of Delta cases; odds ratio, 1.93; 95% CI 1.88–1.98). On the other hand, a loss of smell and taste was found to be less common in subjects infected with Omicron, compared with Delta cases (13% of Omicron cases, 34% of Delta cases; odds ratio, 0.22; 95% CI 0.21–0.23) [11]. In the present study, a sore throat was more frequent in the 6th wave group (60.4%) than in the 1st–5th wave group (10.6%, *p* < 0.0001), which was consistent with the findings for the general population. Olfactory and taste abnormalities tended to be less frequent in the 6th wave group (5.7%) than in the 1st–5th wave group (10.6%), but the difference was not significant. This lack of significance may be partly due to the fact that the study participants were relatively old (olfactory and taste abnormalities are more common in young people) and the number of study participants in our study was somewhat small. Headache was also a less frequent symptom in the 6th wave group (9.4%) than in the 1st–5th wave group (25.5%, *p* = 0.032), which was also consistent with the findings for the general population [11].

The serum albumin level was higher and the serum LDH level was lower in the 6th wave group than in the 1st–5th wave group. Elevated serum LDH levels and decreased serum albumin levels have been reported as risk factors for severe COVID-19 [11]. The reason for this finding in the present study is thought to be that the number of days until blood collection was significantly shorter in the 6th wave group and that the patient outcomes were relatively good. Anticoagulation therapy was used more in the 1st–5th wave group than in the 6th wave group even though the value of D-dinner at the time of admission was not different between the two groups. There were some cases whose level of D-dinner elevated during the hospitalization (data not shown). Therefore, in the 1st–5th wave group, more patients underwent anticoagulant therapy than in the 6th wave group even though the value of D-dinner at the time of admission was not different.

In the general population, a stratified Cox proportional hazard regression analysis suggested that the risk of presentation to emergency care or hospital admission after infection with Omicron was approximately three-fifths of that for Delta (hazard ratio, 0.62; 95% CI 0.55–0.69) [12]. In the present study, none of the patients in the 6th wave group developed the critical disease, while 21.3% of the patients in the 1st–5th wave group reached a critical stage (*p* < 0.001). Fewer hemodialysis patients in the 6th wave group required steroid (5.7%) or anticoagulant (13.2%) therapy, compared with those in the 1st–5th wave group (53.2%, *p* < 0.0001 and 46.7%, *p* < 0.001, respectively). The number of days from onset until a reduction in fever was shorter in the 6th wave group (6.1 ± 4.6 days) than in the 1st–5th wave group (9.5 ± 3.0 days, *p* = 0.012). The number of days from onset until hospital discharge was also shorter in the 6th wave group (11.5 ± 3.3 days) than in the 1st–5th wave group (18.1 ± 7.2 days, *p* < 0.0001). The maximum CRP level during hospitalization was lower in the 6th wave group [1.99 (0.57–6.24) mg/dL] than in the 1st–5th wave group [10.42 (5.22–14.12) mg/dL, *p* < 0.0001]. The cutoff value for the maximum CRP value within 7 days after clinical onset for predicting a critical outcome was 9.34 mg/dL in the 1st–5th wave group, with a sensitivity of 100% and a specificity of 79%. The area under the ROC curve was 0.89. A rapid increase in the serum CRP to a level over 9.34 mg/dL within 7 days after clinical onset may have been an important sign of a potentially critical outcome in the 1st–5th wave group. In the 6th wave group, 7 hemodialysis patients had CRP values above 9.34 mg/dL, but none of these patients developed critical outcomes. The maximum CRP during hospitalization was relatively low in the 6th wave group, and even in 6th wave patients with an elevated CRP level, none of them developed critical disease. In addition to the attenuated virus, the reason for the relatively good outcomes in the 6th wave group may be partly attributable to the high vaccination rate (2 doses or more, 96.2%; 3 doses, 18.9%), compared with the 1st–5th wave group. A study from Scotland revealed that the mortality of patients with kidney failure who developed COVID-19 was 22.5% before vaccination, with this figure decreasing to 9.2% after two vaccine doses [13]. Another reason for the relatively good outcome in the 6th wave group might be the advances in the treatment of COVID-19 that had become available. Neutralizing antibody therapy was used more frequently in the 6th wave group (75.5%) than in the 1st–5th wave group (14.9%, *p* < 0.0001). It has been reported that casirivimab and imdevimab have a reduced neutralizing activity against Omicron strains [10]; they were not used in the 6th wave group. Instead, sotrovimab was used only in the 6th wave group. The virus tends to mutate, and Casirivimab/Imdevimab, which was effective during the 5th wave (Delta variant), is less effective in the 6th wave (Omicron variant). Furthermore, BA.1 has been replaced by BA.2 in Omicron strains, and the effect of sotrovimab has been weakened.

Molnupiravir also became clinically available in Japan during the 6th wave of COVID-19. Molnupiravir was used in 88.7% of the patients in the 6th wave group, but could not be used in any of the patients in the 1st–5th wave group.

The limitations of this study were that it was designed as a retrospective cohort study, that all the study participants were from a single institution, and that the sample size was limited. To identify the prognosis factors of the disease severity for COVID-19, multivariate analyses are required. But we could not perform cause of small size of patients. We also could not perform ROC curve analysis to determine the optimum cutoff point for the maximum CRP for predicting a critical outcome in the 6th wave group because there were no patients who result in critical in the group.

## Conclusion

The outcomes of hemodialysis patients in the 6th wave group were better than those in the 1st–5th wave group. In addition to the attenuated pathogenicity of the SARS-CoV-2 Omicron variant, the high prevalence of vaccination in the 6th wave group and advances in the treatment of COVID-19, such as neutralizing antibody therapy or antiviral drugs, may have contributed to these good outcomes.

## Data Availability

The datasets during and/or analyzed during the current study are available from the corresponding author upon reasonable request.

## Abbreviations

COVID-19: Coronavirus disease 2019
SARS-CoV-2: Severe acute respiratory syndrome coronavirus 2
RT-PCR: Reverse transcription polymerase chain reaction
SpO2: Oxygen saturation level
CRP: C-reactive protein
ROC: Receiver operating characteristic
TP: Total protein
LDH: Lactate dehydrogenase
